# Lactylation of PFKP-K688 enhances glycolytic flux and confers cardioprotection in myocardial ischemia

**DOI:** 10.3389/fphar.2026.1717779

**Published:** 2026-03-16

**Authors:** Cui Liu, Lujing Jiang, Panxia Wang, Yuehuai Hu, Yongjia Zheng, Lingzi Lu, Jing Lu, Yang Mao, Peiqing Liu

**Affiliations:** 1 National and Local Joint Engineering Lab of Druggability and New Drugs Evaluation, Guangdong Provincial Key Laboratory of New Drug Design and Evaluation, Guangdong Province Engineering Laboratory for Druggability and New Drug Evaluation, School of Pharmaceutical Sciences, Sun Yat-sen University, Guangzhou, China; 2 School of Materials Science and Engineering, Sun Yat-sen University, Guangzhou, China; 3 Core Laboratory, Fujian Medical University Clinical Teaching Hospital, Fuzhou Pulmonary Hospital of Fujian Province, Fuzhou city, Fujian, China; 4 Guangzhou Municipal and Guangdong Provincial Key Laboratory of Molecular Target & Clinical Pharmacology, The NMPA and State Key Laboratory of Respiratory Disease, School of Pharmaceutical Sciences, Guangzhou Medical University, Guangzhou, China

**Keywords:** glycolysis, lactic acid, lactylation, myocardial ischemia, PFKP

## Abstract

Myocardial ischemia triggers metabolic reprogramming characterized by enhanced glycolysis and accumulation of lactic acid. However, the functional relevance of lactic acid-induced glycolysis-related protein lysine lactylation (Kla) remains poorly understood. In this study, utilizing both *in vivo* (LAD-operated mice) and *in vitro* (hypoxic AC16 cardiomyocytes) models, we have identified PFKP-K688 site lactylation (PFKP-K688la) as a crucial metabolic regulatory target in myocardial hypoxia. Using lactylome proteomics, we identified 521 Kla modified proteins, among which PFKP emerged as the primary target. Although PFKP protein levels remained unchanged under hypoxic conditions, its K688la modification increased, resulting in enhanced enzymatic activity. This modification increased glycolytic ECAR flux while concurrently suppressing mitochondrial respiration OCR. Nala elevates PFKP-Kla to rescue cardiomyocytes from hypoxic injury, thereby enhancing cell survival, restoring contractility, and reversing 2-DG-induced toxicity. Furthermore, the PFKP-K688E mutation, mimicking hyper-lactylation, further amplifies glycolysis. Our findings reveal a positive feedback loop in which hypoxia-induced lactate production promotes PFKP-K688 lactylation, thereby boosting glycolytic output and conferring cytoprotection, which may be a therapeutic target for ischemic cardiomyopathy.

## 1 Introduction

Cardiovascular disease (CVD) is the leading cause of death and disability worldwide, accounting for approximately 25%–30% of total global mortality (Ganzer et al., 2022). Myocardial ischemia is one of the core pathological features of CVD (Ardehali and Ports, 1990; Hinderliter et al., 1991; Deedwania and Carbajal, 1992), which is caused by myocardial insufficiency caused by coronary artery occlusion or stenosis, and its essence is the imbalance between myocardial oxygen supply and oxygen demand. Persistent or severe ischemia can impair myocardial cell function, leading to cardiac insufficiency, arrhythmia, myocardial infarction, sudden death and heart failure (Shimokawa and Yasuda, 2008; Ahmad et al., 2022).

Healthy adult cardiac myocytes primarily rely on mitochondrial oxidative phosphorylation as their main energy source (Lehman et al., 2000). However, during myocardial ischemia, reduced coronary blood flow leads to energy and nutrient deprivation as well as hypoxia (Wu et al., 2023; Kierans and Taylor, 2024), which in turn triggers a compensatory surge in glycolysis (Webster, 2003). While this evolutionarily conserved response rapidly generates ATP to support cell survival under hypoxic conditions, it also causes excessive lactic acid accumulation, leading to intracellular acidosis, disrupted calcium homeostasis, and impaired contractile function (Neely and Grotyohann, 1984; Lu et al., 2021).

Lactic acid, conventionally viewed as a metabolic byproduct of glycolysis and a potential agent of cellular damage (Chen et al., 2021), was recently discovered to possess a novel biological role by Zhao’s team in 2019 (Zhang et al., 2019). Specifically, lactic acid can induce lysine Kla by forming covalent bonds with lysine residues on specific proteins (Li H. et al., 2024). This unique post-translational modification modulates protein activity through allosteric mechanisms, influencing signal transduction and metabolic control (Wang et al., 2022). The involvement of lactic acid modification in cardiovascular conditions has garnered significant interest. Research indicates that histone lactylation can regulate the anti-inflammatory and proangiogenic functions of monocytes and macrophages (Wang et al., 2022), facilitate tissue repair, and enhance cardiac performance post-myocardial infarction. Furthermore, tumor necrosis factor receptor-associated protein 1 (TRAP1) promotes smooth muscle cell senescence via HDAC3-mediated histone lactylation, exacerbating atherosclerosis (Li X. et al., 2024), while Snail1 lactylation drives endothelial-mesenchymal transition following myocardial infarction (Fan et al., 2023). Notably, mice lacking the α-myosin heavy chain (α-MHC) K1897 lactase site exhibit compromised α-MHC interaction with Titin proteins, leading to severe heart failure (Zhang et al., 2023). Despite these findings, the comprehensive functional network of lactate modification in ischemic cardiomyocytes, particularly its precise regulatory mechanisms on key energy metabolism enzymes, necessitates further elucidation.

Fructose-6-phosphokinase-1 (PFKP) serves as the principal rate-limiting enzyme in the glycolytic pathway by catalyzing the irreversible conversion of fructose-6-phosphate (F6P) to fructose-1,6-bisphosphate (F1,6BP). This enzymatic reaction represents a critical control point that governs the flux of glycolysis (Mor et al., 2011; Lee et al., 2017). PFKP orchestrates the partitioning of glucose carbon flux between glycolysis and alternative pathways in response to cellular energy status via allosteric regulation. Recent investigations have elucidated that PFKP can additionally coordinate metabolic and disease-associated signaling cascades by undergoing dynamic alterations in subcellular localization (Broxmeyer, 2021), diverse post-translational modifications (Lee et al., 2018), and epigenetic control mechanisms (Liu L. et al., 2024). Notably, PFKP plays a pivotal role in the pathogenesis and progression of various disorders, encompassing cancer (Liu W. et al., 2024) and cardiovascular diseases (Vigil-Garcia et al., 2021).

This study focuses on the regulatory effect of lactylation-modification on myocardial ischemic energy metabolism remodeling. For the first time, we found that PFKP at K688 is a critical lactylation-modification site in hypoxic cardiomyocytes. After hypoxia stimulation, PFKP-K688la level increased significantly and directly affected PFKP enzyme activity. This study aims to systematically clarify the central role of PFKP-K688 lactylation-modification in regulating energy metabolism under myocardial hypoxia, and to provide a solid theoretical basis and experimental basis for further understanding the pathophysiological mechanism of ischemic heart disease and developing new therapeutic strategies targeting metabolic remodeling.

## 2 Materials and methods

### 2.1 Reagents and antibodies

Isoflurane was purchased from Ezvet (China). Bcl2 (Proteintech Cat# 26593-1-AP, RRID: AB_2818996), Bax (Proteintech Cat# 50599-2-Ig, RRID: AB_2061561), PFKP (Proteintech Cat# 13389-1-AP, RRID: AB_2252278), α-tubulin (Proteintech Cat# 11224-1-AP, RRID: AB_2210206) antibody, mouse secondary antibody (Proteintech Cat# SA00001-1, RRID: AB_2722565) and rabbit secondary antibody (Proteintech Cat# SA00001-4, RRID: AB_2864335) were purchased from Proteintech Group, Inc. (China). Reverse transcription and SYBR reagents were purchased from Vazyme Biotech Co.,Ltd. (China). Coomassie Brilliant Blue G-250 was purchased from Macklin (China). Anti-lactyl lysine rabbit antibody was purchased from PTM Biolabs Inc. (CAT# PMT-1401) (China). TMT-6 (CAT# 90060), Pancreatin (CAT# 90058), DTT (CAT# D1532), and IAA (CAT# A39271) were purchased from ThermoFisher Scientific (USA). Nala, DCA, Oxamate and 2-DG were purchased from Med Chem Express (USA). Type I murine tail collagen was purchased from Solarbio Science and Technology Co.,Ltd. (CAT# C8062) (China). CCK8 and IgG were purchased from Beyotime Biotechnology (China). AC16 cells (RRID:CVCL_4U18) were purchased from ATCC (USA). Cell culture medium was purchased from GE Hyclone (CAT# SH30022.01) (USA). Cell lysis buffer was purchased from Waters (CAT# 186001861) (USA). L-Lactic Acid content assay kit was purchased from BOXBIO (CAT# AKAC001M) (China). PFKP activity detection kit was purchased from BOXBIO (CAT# AKSU062M) (China).

### 2.2 Animals

Male C57BL/6J mice (6–8 weeks old) were obtained from Zhuhai Best Biotechnology Co., Ltd. All animals were maintained in the specific pathogen-free (SPF) facility of the Laboratory Animal Center, Sun Yat-sen University, under strictly controlled environmental conditions: temperature at 22 °C ± 1 °C, relative humidity of 40%–50%, and a 12-h light/dark cycle. Mice had *ad libitum* access to sterile drinking water and standard laboratory chow. All animal experimental procedures were performed in compliance with the Guide for the Care and Use of Laboratory Animals published by the National Institutes of Health (NIH) and were approved by the Institutional Animal Care and Use Committee of Sun Yat-sen University. Before sample collection, mice were anesthetized with 2% isoflurane and humanely euthanized by cervical dislocation. No mice were excluded from the study except for the surgical failure. The mice were anesthetized with 2% isoflurane before sampling and were euthanized for posterior cervical dislocation.

### 2.3 Mice LAD model

A myocardial infarction model was induced in C57BL/6J mice via left coronary artery ligation. Mice were initially anesthetized with 2% isoflurane delivered at a fresh gas flow of 4L/min (0.41 mL/min isoflurane) and continuously ventilated via a breathing mask. Maintained on a heating plate to preserve body temperature, they underwent chest opening at the third intercostal space above the left thorax for heart exposure, followed by ligation of the left anterior descending (LAD) artery using 6–0 silk thread.

In establishing a mouse model of myocardial ischemia via LAD surgery, the mortality rate predominantly occurs within 24–48 h post-operation. The primary causes of mortality include: I. Acute malignant arrhythmia; II. Anesthesia-related incidents, pneumothorax, or postoperative respiratory failure. The study involved 8 mice in the sham group and 8 mice in the ischemia group, with 2 mice succumbing in the ischemia group. The electrocardiograms of the surviving mice in the ischemia group exhibited ST-segment elevation, confirming successful ligation. Consequently, the remaining 6 mice were utilized for cardiac ultrasound and additional experiments, consistent with the mortality rate for this mouse model typically ranges from 20% to 40%.

The exclusion criteria are as follows: I. Mice that died within 24–48 h post-operation were included in the mortality statistics and excluded from functional endpoint analysis; II. Mice exhibiting no ST-segment elevation on electrocardiograms following LAD surgery; III. Animals in the sham operation control group experienced myocardial injury.

### 2.4 Culture of AC16 cardiomyocyte cell lines

Cardiomyocytes were placed in a double-antibody-containing high-glucose medium containing 10% fetal bovine serum (Fetal Bovine Serum) in a cell room incubator at 5% CO2 and 37 °C.

### 2.5 Establishment of myocardial oxygen glucose deprivation (OGD) model

After aspirating the normal medium, wash it with PBS. The blank group was incubated with high-glucose serum-free medium in a 5% CO2 incubator at 37 °C, while the hypoxia group was incubated with sugar-free serum-free medium in a 1% O2 incubator.

### 2.6 Echocardiography

Place the mice in an anesthesia induction box, introduce 2%–3% isoflurane (oxygen flow rate 0.5–1L/min), and after the limbs relax, move them to a heating pad (maintain body temperature at 37 °C ± 0.5 °C), fix the limbs, connect the electrocardiogram electrodes, and maintain 1%–2% isoflurane anesthesia. Chest hair removal was performed, coupling agent was applied, the probe was gently placed on the left edge of the sternum, the short-axis section was switched, M-mode ultrasound was obtained, and the ejection fraction and shortening rate were calculated. Stop the anesthesia. After the animal resumes its independent activities, put it back into the cage and monitor its breathing and heart rate throughout the process.

### 2.7 HE staining

Tissue sections were baked at 60 °C for 30 min, then dewaxed in xylene I and II (10 min each). They were immersed in absolute ethanol I and II (5 min each), followed by gradient rehydration in 95%, 80%, 70% ethanol (3 min each) and rinsed with distilled water for 2 min. Sections were stained with hematoxylin (5–10 min), rinsed to remove excess dye, differentiated with 1% hydrochloric acid-ethanol (30 s), then rinsed for 15 min to blue. After eosin staining (30 s-1 min) and a brief distilled water rinse, they were dehydrated in 70%, 80%, 95% ethanol I (30 s each), followed by 95% ethanol II, absolute ethanol I and II (5 min each). Cleared in xylene I and II (10 min each), air-dried briefly, mounted with neutral gum, covered with coverslips (avoiding bubbles), and dried in a well-ventilated area.

### 2.8 Western blotting

After subculture, drug administration and cultivation of AC16 cells, cellular proteins were extracted, and gels and electrophoresis buffers were prepared as required. Subsequently, sample loading and gel electrophoresis were performed, with stepwise electrophoresis at specified voltages. Relevant electrotransfer operations were then conducted. Following overnight incubation with primary antibodies, the corresponding mouse or rabbit secondary antibodies were applied for 2 h at room temperature. Finally, chemiluminescent detection reagents were used for development, and images were recorded and saved.

### 2.9 Quantitative real-time PCR

Fresh samples were lysed with Trizol. Chloroform was added, vigorously vortexed for 15s, and incubated at room temperature for 3min, followed by centrifugation at 12,000×g for 15min at 4 °C. The upper aqueous phase was transferred to a new tube, mixed with equal-volume isopropanol, incubated at room temperature for 10min, and centrifuged at 12,000×g for 10min at 4 °C. The RNA pellet was retained, washed with 75% ethanol, air-dried at room temperature, and dissolved in RNase-free water.

After qualifying RNA concentration and purity, 1 μg RNA was used for cDNA synthesis via reverse transcription with primers and enzymes per the kit instructions on a PCR thermocycler. Using cDNA as a template, qPCR was performed with specific primers and master mix under predefined protocols. Amplification and melting curves were analyzed, and relative mRNA expression was calculated via standard curves. The primers are shown in Table 1.

**TABLE 1:** List of primers for qPCR analysis.

Genes (Rat)	Primer (Forward)	Primer (Reverse)
*PFKP*	AGC​TTG​CGT​CGT​GTC​ACT​GAA​C	ATC​TCC​TCT​CGT​CCA​TCG​CCT​TC
*ALDOA*	GGG​CTC​CCT​CCC​CAT​CAA​TA	TAG​GGA​AAC​CTG​AAG​CCC​CT
*LDHA*	ACG​TGC​ATT​CCC​GAT​TCC​TT	AAC​AGC​ACC​AAC​CCC​AAC​AA
*PKM*	TCT​CTT​CGT​CTT​TGC​AGC​GT	CTC​TGA​GCT​CCA​CTG​CAT​CC
*Eno1*	CCT​GCC​CTG​GTT​AGC​AAG​AA	GGC​GTT​CGC​ACC​AAA​CTT​AG
*β-actin*	GGC​CAA​CCG​CGA​GAA​GAT​GAC	GGA​TAG​CAC​AGC​CTG​GAT​AGC​AAC

Genes (*Mouse*)	Primer (Forward)	Primer (Reverse)
*PFKP*	CCC​ATG​GTT​ATG​GTT​CCT​GCT	GGT​CGC​ACG​TGT​CTG​TGA​TA
*ALDOA*	AGC​TGA​ATA​GGC​TGC​GTT​CT	GAC​AGG​CGG​GTC​ATG​TTG​AA
*PKM*	GCT​CTA​GGT​ATC​GCA​GCA​GG	TGT​TCC​AGG​AAG​GTG​TCA​GC
*Eno1*	AAA​GGT​CTC​TTC​CGA​GCT​GC	GCT​GGT​GCC​AGG​AAC​TCA​TT
*TPI*	GTC​AAT​GAT​GGG​GTG​GCT​CA	GCA​GTG​CTC​ATT​GTT​TGG​CA
*GPI*	GGG​ACC​CAC​ATT​GCC​AAA​AC	CGC​TTC​GAG​AAA​CCA​CTC​CT
*HEXO*	GTG​ATG​GTG​GTG​GTG​GTA​GG	CAC​CCC​AAG​GAA​ACA​CCA​CT
*PGK*	GGT​GTT​GCC​AAA​ATG​TCG​CT	GGA​CTT​GGC​TCC​ATT​GTC​CA
*β-actin*	TGA​GCT​GCG​TTT​TAC​ACC​CT	GCC​TTC​ACC​GTT​CCA​GTT​TT

### 2.10 Lactic acid detection

First, prepare cell or tissue samples; then add extraction buffer as instructed by the kit, mix well for lysis, and subsequently centrifuge to collect the supernatant. Next, dilute the standards to make gradient solutions, and then transfer the sample supernatant and gradient standards into reaction wells, followed by adding the detection reagent; after mixing, incubate at 37 °C for 30 min. Then, use a microplate reader to measure the absorbance at 450nm, and finally draw a standard curve to calculate the lactic acid concentration of the samples based on it.

### 2.11 Lactylation proteomics analysis

Take fresh tissue or cell samples, wash twice with pre-cooled PBS, add 50 mM ammonium bicarbonate buffer containing 0.5% RapiGest SF Surfactant (Waters, Milford, MA), sonicate and lyse on ice for 30 min. Centrifuge at 12,000 g at 4 °C for 15 min and take the supernatant. Measure the protein concentration by the Comax-brilliant blue method. Proteins were reduced with 10 mM DTT for 45 min at 60 °C and then alkylated with 20 mM iodoacetamide for 30 min at room temperature in the dark, then trypsin at a ratio of 1:100 and enzymatized at 37 °C overnight. Digestion was stopped by adding trifluoroacetic acid (TFA) until the pH was 2. The enzymatic hydrolysis products were desalted using reverse-phase Sep-Pak C18 cartridges (Waters, Milford, MA) and eluted with 50% methanol, 0.1% formic acid. Peptides were quantified using Pierce Quantitative Colorimetric Peptide Assay (Thermo, 23275). peptide samples were separately labeled using TMT 6Plex (Thermo, 90066), in 100 mM TEAB buffer according to the manufacturer’s recommendations. The specific antibody for lactic acid modification was incubated with protein A/G magnetic beads at 4 °C for 2 h to prepare the antibody-magnetic bead complex. The TMT-labeled peptides were added and incubated overnight at 4 °C to enrich the lactic acid peptide segment. The magnetic beads were washed three times with washing buffer and eluted with an acetonitrile solution containing 5% ammonia water. The eluate was vacuum-dried and then reconstituted. The enriched Kla peptides were dissolved in 0.1% (v/v) formic acid (solvent A) and loaded onto an in-house reversed-phase analytical column (C18-AQ, 1.9 μm, PF360-75-10-N-5, 20 cm length). The solvent gradient consisted of an increase in the range of 3%–32% solvent B (0.1% (v/v) formic acid in 80% (v/v) acetonitrile) over 95 min, an increase in the range of 32%–100% over 10 min, and a final 100% over 15 min at a constant 200 nL/min flow rate in an EASY-nLC 1200 UPLC system (Thermo Fisher Scientific). The peptides were subjected to the Nano Spray Ionization (NSI) source followed by tandem mass spectrometry (MS/MS) in a QExactivePlus (ThermoFisher Scientific) coupled online to the UPLC. The electrospray voltage was 2.1kV, the m/z full scan range was 355–1,700, and the intact peptides were detected in an Orbitrap at 70,000 resolutions. The peptides were selected for MS/MS using a normalized collisional energy of 35%, and the fragments were detected in the Orbitrap at 17,500 resolution. The data-dependent procedure alternated between 1 MS scan followed by 10 MS/MS scans with 60.0 s dynamic exclusion. The automatic gain control and the fixed first mass were set to 5E4 and 120 m/z, respectively. The resulting MS/MS data were processed using Protein Discovery 2.2.0.388 (Thermo Fisher Scientific). Tandem mass spectra were searched against the UniProtKB database *Homo sapiens* (SwissProt TaxID 9606) concatenated with a reverse decoy database. Trypsin was the cleavage enzyme that allowed 2% missing cleavages. Mass errors of fragment ions and precursor were set as 0.02 Da and 20 ppm, respectively. Carbamidomethyl on Cys was the fixed modification, and lactylation on Lys and oxidation on Met were the variable modifications. The minimal peptide was set to 5, and the false discovery rate (FDR) was adjusted to <1%. The Kla site localization probability of <0.75 was excluded.

### 2.12 Bioinformatics analysis

Based on the data of lactated modified proteins obtained by mass spectrometry detection, a reliable protein list and corresponding IDs were first sorted out. GO enrichment analysis was conducted through the Metascape database. The P-value and FDR threshold were set to screen for significantly enriched biological processes, cell components, and molecular function items, and a bar chart or bubble chart was generated for display. Then, KEGG enrichment analysis was conducted using the same database to explore the signaling pathways involved in proteins. Key pathways were screened through enrichment factors and P-values, and the pathway network diagram was drawn. For PPI analysis, the protein ID is imported into the STRING database, the species source and interaction score thresholds are set, the protein interaction relationship data is obtained, and the Cytoscape software is used for visualization. The core functional modules are identified through the MCODE plugin, and parameters such as node degree and betweenness centrality are calculated to screen key proteins.

### 2.13 Immunoprecipitation-mass spectrometry (IP-MS)

AC16 cells from different groups were harvested, and total proteins were extracted and quantified using a bicinchoninic acid (BCA) assay. For immunoprecipitation, 500 μg aliquots of total protein were incubated with PFKP primary antibodies at 4 °C overnight with gentle shaking. Subsequently, protein A/G magnetic beads were added, and the mixture was further incubated at 4 °C for 4 hours to form antigen-antibody-bead complexes. The magnetic beads were washed five times with pre-cooled wash buffer, with each wash followed by centrifugation at 4 °C for 5 minutes and careful aspiration of the supernatant. After the final wash, elution buffer was added and incubated at room temperature for 10 minutes to elute the target proteins. The immunoprecipitated proteins were separated by 10% Polyacrylamide Gel Electrophoresis (PAGE) and visualized by Coomassie Brilliant Blue R-250 staining. Differential PFKP-containing gel bands were excised under sterile conditions for in-gel digestion. The in-gel digestion protocol was performed as follows: Gel bands were cut into 1 mm^3^ fragments and destained with 50 mM NH_4_HCO_3_ containing 30% acetonitrile (ACN) until the gel pieces were transparent. The destained gel pieces were then reduced with 300 μL of 10 mM dithiothreitol (DTT) in 50 mM NH_4_HCO_3_ at 60 °C for 1 hour, followed by alkylation with 60 mM iodoacetamide in 50 mM NH_4_HCO_3_ in the dark at room temperature for 45 minutes. After alkylation, the gel pieces were incubated with 80 μL of 50 mM NH_4_HCO_3_ containing 2 μg of trypsin at 37 °C overnight (16 hours) for digestion. The digested peptides were extracted from the gel fragments using ACN containing 0.1% formic acid (FA), and the extracts were pooled. The peptide mixture was desalted using a C18 solid-phase extraction column, vacuum-dried, and reconstituted in 0.1% FA. Finally, the reconstituted peptides were analyzed and identified by liquid chromatography-tandem mass spectrometry (LC-MS/MS).

### 2.14 Extracellular acidification rate (ECAR) and oxygen consumption rate (OCR)

ECAR detection: Take logarithmic phase cells, adjust the concentration, and then inoculate them into a dedicated detection plate. Incubate at 37 °C overnight. Incubate with sugar-free detection medium for 1 h, place in the Seahorse instrument, and successively inject glucose, oligomycin, and 2-deoxyglucose. Record the changes in extracellular acidification rate. The instrument automatically calculates indicators such as basic glycolysis, glycolysis capacity and reserve, sets blank Wells to correct the background, and the data is statistically analyzed after normalization processing.

OCR detection: Cell inoculation was the same as ECAR, and the cells were incubated in serum-free detection medium for 1 h. After being placed in the instrument, oligomycin, FCCP, and antimycin A/rotenone were injected successively to monitor the oxygen consumption rate. The instrument calculated indicators such as basic respiration, ATP generation, maximum respiration and spare respiratory capacity, set the blank well correction background, and the data were statistically analyzed after normalization processing.

### 2.15 Collagen cell contraction assay

Digest the cells and mix them in a ratio of collagen solution: 10× culture medium: sterile water: cell suspension = 7:1:1:1. Adjust the pH to 7.2–7.4 with 0.1M NaOH and gently pipette to avoid air bubbles. Take 200 μL of the mixed solution from each well and add it to a 24-well plate. Let it stand in a 37 °C incubator for 30 min until the gel solidifies. Carefully add 1 mL of complete culture medium to avoid dispersing the gel. Observe the gel shrinkage every day. The experiment was repeated three times, and the gel area was measured by ImageJ software to calculate the shrinkage rate.

### 2.16 Coomassie brilliant blue detection

Dissolve Coomassie Brilliant Blue G-250 in methanol - glacial acetic acid - aqueous solution (in a ratio of 4:1:5), stir until completely dissolved, then filter and set aside. Slowly add the staining solution to the gel surface, ensuring that the liquid level covers the gel by approximately 1 cm. Let it stand at room temperature for staining for 30 min. After staining is completed, wash the gel with the decolorizing solution until the background color of the gel fades and the target band is clear.

### 2.17 Statistical analysis

All data are expressed as mean ± SD. For data meeting the assumptions of normality and homogeneity of variance, comparisons between two groups were performed using an unpaired two-tailed Student’s t-test. For multiple group comparisons, one-way ANOVA followed by Tukey’s multiple comparisons test was applied. A P-value <0.05 was regarded as statistically significant. All statistical analyses were carried out using GraphPad Prism 9.0 software.

## 3 Results

### 3.1 PFKP mRNA expression is reduced in myocardial ischemic mice and hypoxic cardiomyocytes

**FIGURE 1:**
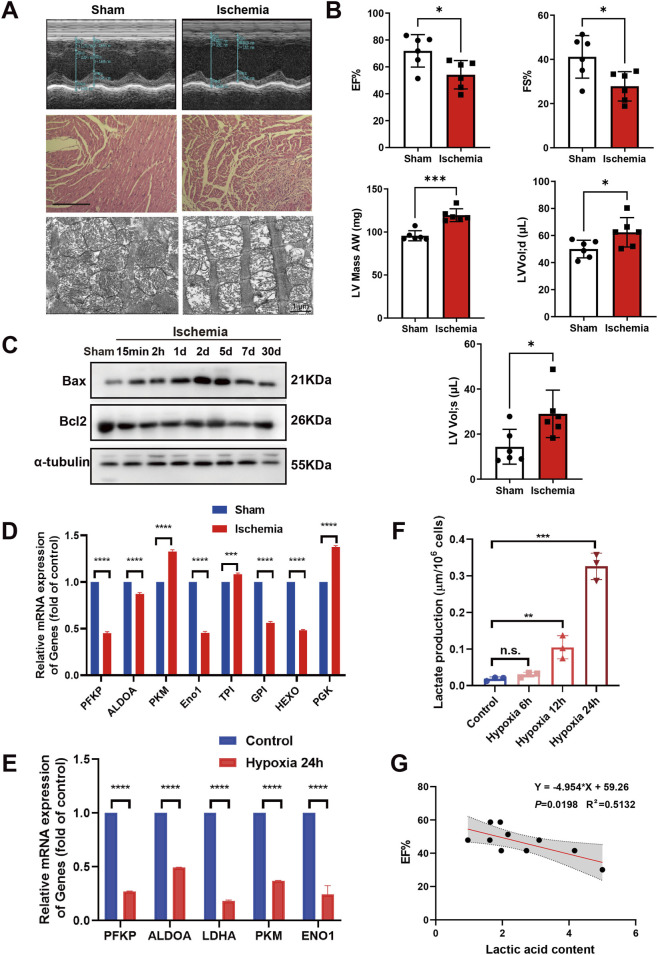
Detection of PFKP protein expression level. **(A)** The upper panel displays mouse echocardiography, and the middle panel exhibits HE staining. Scale bar, 125μm; the lower panel exhibits the mitochondria under the electron microscope. Scale bar, 1 μm. **(B)** Echocardiographic assessment of cardiac function in mice (n = 6) included measurements of EF% (ejection fraction), FS% (fractional shortening), LV Mass AW (left ventricular anterior wall mass), and LV Vol; d/s (left ventricular diastolic/end-systolic volume). **(C)** Western blot analysis was utilized to assess Bax/Bcl-2 protein expression at various time points following myocardial ischemia, with α-tubulin serving as an internal reference (n = 3). **(D,E)** Evaluation of mRNA levels of glycolysis-related proteins in LAD mice and hypoxic AC16 cells was conducted (n = 3). PFKP: phosphofructokinase, platelet; ALDOA: aldolase, fructose-bisphosphate A; PKM: pyruvate kinase M1/2; Eno1:enolase 1; TPI Triose phosphate isomerase; GPI: glucose-6-phosphate isomerase; HEXO: Hexokinase; PGK: Phosphoglycerate kinase; LDHA: lactate dehydrogenase **(A)**. **(F)** Assessment of lactic acid content was performed (n = 3). **(G)** EF-serum lactic acid content correlation chart (n = 10). Statistical significance was calculated using an unpaired two-tailed Student’s t-test or one-way ANOVA. Data are shown as mean ± SD. ****P < 0.0001; ***P < 0.001; **P < 0.01; *P < 0.05, ns means no significance.

In a study where the left anterior descending coronary artery (LAD) was ligated in mice to induce myocardial ischemia for 2 days, various parameters were assessed. Echocardiography showed a decrease in ejection fraction (EF) and fractional shortening (FS) after the procedure, indicating reduced cardiac pumping ability and a decline in cardiac function. There was also an increase in left ventricular (LV) mass, LV volume in diastole (LV Vol; d), and LV volume in systole (LV Vol; s) (Figures 1A,B), suggesting left ventricular hypertrophy, increased preload, and impaired systolic function. Histological analysis with hematoxylin and eosin (HE) staining confirmed disorganized myocardial cells and increased inflammatory infiltration in ischemic heart of mice, confirming successful induction of ischemia (Figures 1A,B). We observed that mitochondria in the ischemic group showed significant swelling throughout. Their internal cristae were disrupted, dissolved, and disordered in arrangement, with a significant reduction in cristal density, accompanied by sparse and vacuolated matrix, indicating that ischemic injury led to severe mitochondrial structural damage (Figure 1A). Additionally, levels of the mitochondrial apoptosis protein Bax/Bcl2 rose with longer ischemia duration and returned to baseline by day 30 (Figure 1C), indicating mitochondrial involvement in apoptosis during ischemia.

Ischemia and hypoxia are known to be closely associated with glycolysis. Normally, cells primarily rely on aerobic respiration for energy production, which is highly efficient. However, during ischemic and hypoxic conditions where oxygen supply is limited, aerobic respiration is impeded due to the lack of oxygen, leading to glycolysis becoming the predominant energy generation pathway. Despite its lower efficiency, glycolysis can swiftly generate a modest amount of ATP to sustain fundamental cellular functions. Our investigation into the gene expression of glycolysis-related enzymes in ischemic mice and hypoxic cardiomyocytes revealed a significant abnormal expression pattern (Figures 1D,E). PFKP, ALDOA, and ENO1 are three key enzymes in the glycolysis pathway and significantly decrease in during ischemic (Figures 1D,E). Intracellular lactate levels in AC16 cardiomyocytes increased progressively with the duration of hypoxia (Figure 1F). Moreover, we found that the serum lactate level in the ischemic mice was negatively correlated with EF (Figure 1G). These results revealed a closely relationship of the serum lactate level with ischemic heart.

### 3.2 Lactated protein modification histology identifies lactated proteins

**FIGURE 2:**
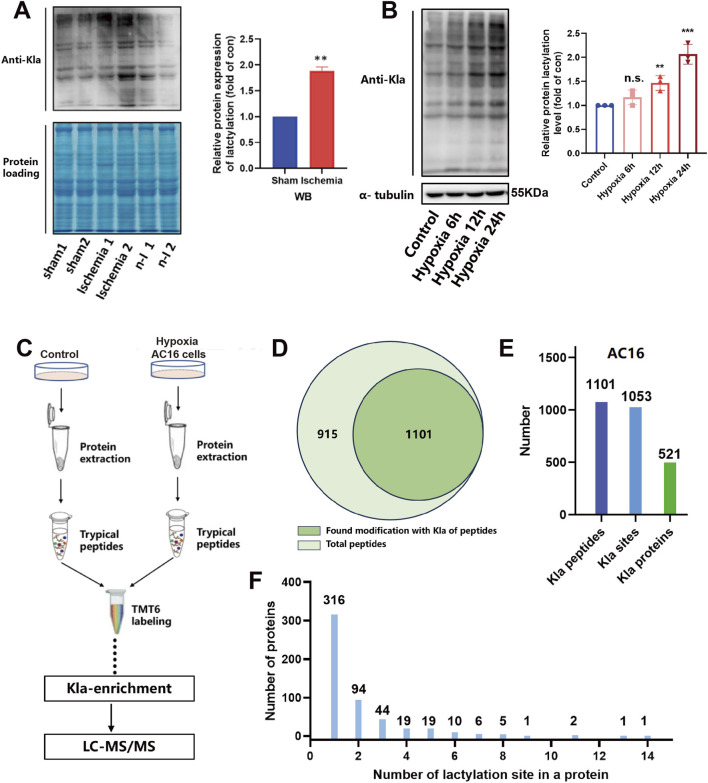
Lactylation proteomics detection. **(A,B)** Pan-Kla antibody detection of lactic modification level of ischemic mouse myocardial tissue and hypoxic cardiomyocytes (n = 3). **(C)** Lactic modification proteomics detection process. **(D–F)** Kla modification on peptides and proteins data analysis. Statistical significance was calculated using an unpaired two-tailed Student’s t-test or one-way ANOVA. Data are shown as mean ± SD. ***P < 0.001; **P < 0.01; ns means no significance.

Research has demonstrated that lactate plays a direct role in regulating protein function through lactylation. This process involves lactate molecules binding to lysine residues on proteins via covalent bonds, subsequently leading to alterations in their conformation and activity. To investigate the impact of lactic acid on the ischemic-hypoxic response of cardiomyocytes via lactylation modification, we observed a notable elevation in lactylation modification levels in cardiomyocytes of ischemic mice (Figure 2A). Moreover, the lactylation modification levels in AC16 cells exhibited a significant increase with the duration of hypoxia (Figure 2B), indicating that the accumulation of lactic acid contributes to heightened protein lactylation modification levels.

To identify proteins exhibiting altered levels of lactate modification, we conducted TMT-6 quantitative labeling on normal and hypoxic 24-h AC16 cell proteins (Figure 2C). The quality control of quantitative proteomics is qualified (Supplementary Figure S1). A total of 2016 peptides were detected, with 1101 peptides displaying lysine lactylation modification. After eliminating duplicate sites, 1053 various amino acid sites exhibited lactylation modification, corresponding to 521 distinct proteins (Figures 2D,E; Supplementary Tables S1-S2). Subsequent analysis revealed that 316 proteins had lactylation modification at a single site, while the remaining proteins displayed modification at multiple sites (Figure 2F).

### 3.3 PFKP is an important lactylation-modifying protein

**FIGURE 3:**
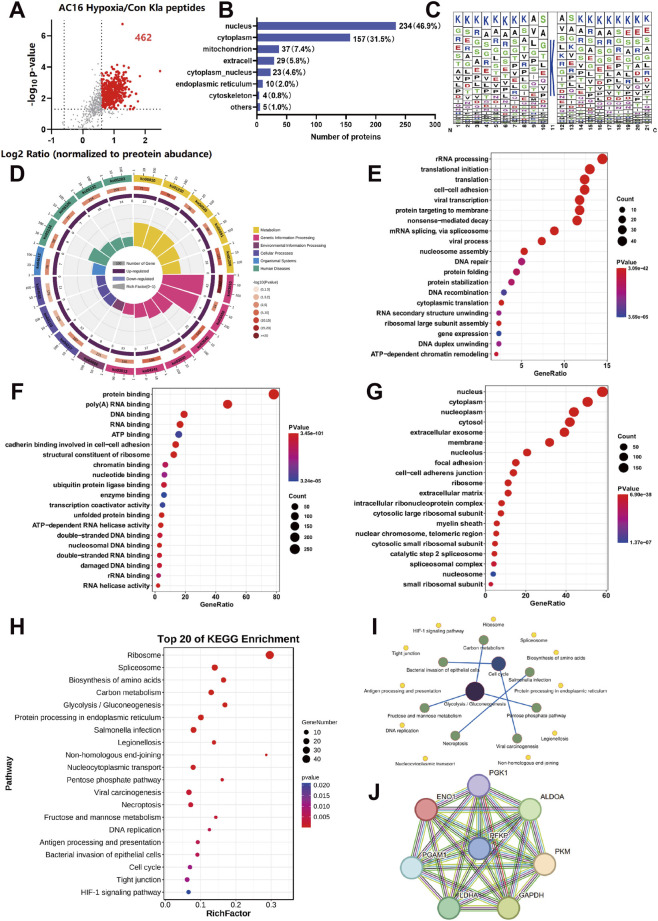
Bioinformatics analysis of lactylation proteomics. **(A)** Volcanic map of Kla-modified peptides. **(B)** Subcellular localization of Kla-modified proteins. **(C)** Sequence Analysis of Kla-modified Peptides. **(D,H)** KEGG Analysis of Kla-modified proteins and elevating the top 20 proteins. **(E–G)** GO enrichment of Kla-modified proteins. **(I)** KEGG interaction network diagram. **(J)** PPI analysis of Kla-modified proteins in the glycolytic pathway.

Bioinformatics analysis revealed that 462 Kla-modified peptides were upregulated in hypoxic AC16 cells (Figure 3A). Of these, 46.9% of Kla proteins were localized in the nucleus, while 31.5% were found in the cytoplasm (Figure 3B). The motif characteristics of these Kla peptides are illustrated in Figure 3C. The elevated Kla-modified proteins were significantly linked to metabolic pathways, such as Glycolysis/Gluconeogenesis, Biosynthesis of amino acids, and Carbon metabolism pathways, as indicated by KEGG circular enrichment maps (Figure 3D). GO enrichment analysis indicated that Kla-modified proteins were primarily involved in rRNA processing, translational initiation, and cell-cell adhesion (Figures 3E–G). They were also found to participate in protein binding and RNA binding functions, predominantly located in the nucleus and cytoplasm (Figures 3E–G). Furthermore, KEGG enrichment analysis of the top 20 proteins highlighted their association with ribosomes, spliceosomes, and biosynthesis of amino acids (Figures 3H,I). Similarly, we discovered the interaction of Kla-modified glycolycle-related proteins (Figure 3J).

Lactylation-modification omics data indicate that in hypoxic AC16 cardiomyocytes, proteins exhibiting upregulated modifications are significantly enriched in the glycolytic pathway. This finding suggests that lactic acid may feedback-regulate glycolysis through post-translational modifications, thereby forming a novel metabolic regulatory circuit. We previously found that the mRNA levels of PFKP, ALDOA and ENO1 were decreased during hypoxia stimulation (Figures 1D,E). PFKP catalyzes the first rate-limiting step, acting like a “metabolic switch”, determining whether glucose can enter the glycolysis pathway, and is precisely regulated by the cellular energy state. Notably, we observed a significant lactylation-modification on the subunit PFKP of phosphofructokinase 1 (PFK-1) with only one modification site identified. This underscores the critical role of lactylation-modification might regulate the function of PFKP. Consequently, we undertook a comprehensive investigation into the function of PFKP.

### 3.4 K688 is an important lactylation modification site for PFKP

**FIGURE 4:**
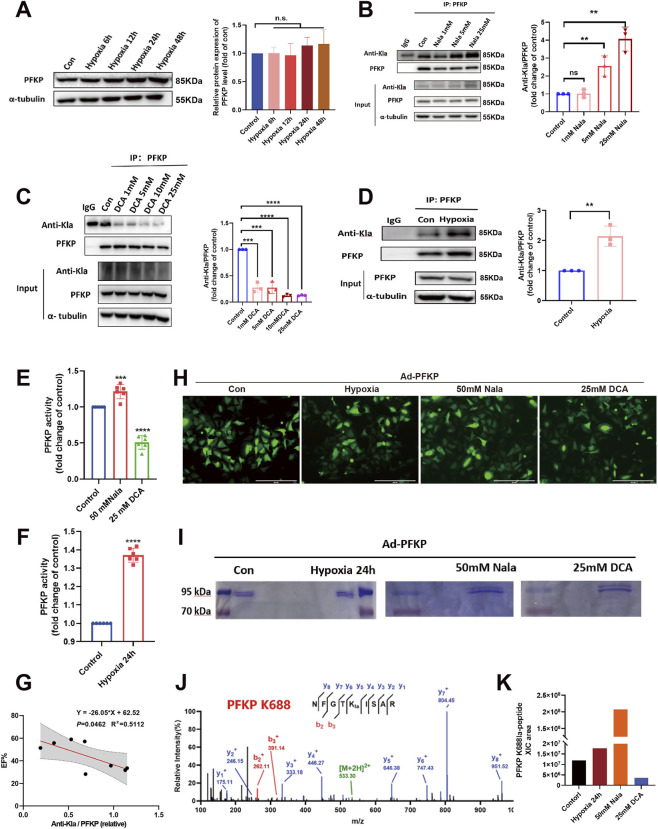
K688 is the only Kla-modified site of PFKP.**(A)** WB detected PFKP protein expression in hypoxic cardiomyocytes (n = 3). **(B–D)** Detection of Kla modification of PFKP in cardiomyocytes under Nala and DCA stimulation and hypoxia (n = 3) **(E,F)** Activity test of PFKP (n = 6). **(G)** EF-PFKP-Kla correlation chart (n = 8). **(H)** Fluorescence detection of the transfection efficiency of Ad-PFKP. Scale bars, 400 μm. **(I,J)** IP-MS identified K688 as the only Kla sites in PFKP. **(K)** XIC area quantifies the PFKP-K688la modification level. Statistical significance was calculated using an unpaired two-tailed Student’s t-test or one-way ANOVA. Data are shown as mean ± SD. ****P < 0.0001; ***P < 0.001; **P < 0.01; ns means no significance.

PFKP protein expression remained constant during hypoxia (Figure 4A). Nala can serve as an exogenous lactate donor, breaking down into lactate^−^ and Na^+^ ions in solution, consequently boosting intracellular lactate levels. To explore the impact of Kla modification on PFKP viability, we supplemented AC16 with Nala, resulting in heightened Kla modification concomitant with increased PFKP activity (Figures 4B,E). Dichloroacetic acid (DCA) inhibits pyruvate dehydrogenase kinase (PDK), leading to the activation of the pyruvate dehydrogenase complex (PDC). PDC is a crucial enzyme in the oxidative metabolism of pyruvate within the mitochondria. Activation of PDC enhances the conversion of pyruvate to acetyl-coenzyme A, consequently reducing the conversion of pyruvate to lactate and lowering lactate levels in the organism. When DCA was introduced to AC16 cells, it resulted in inhibited PFKP-Kla (Figure 4C) and decreased activity of PFKP (Figure 4E). Additionally, under hypoxic conditions, the Kla modification of AC16 was elevated, leading to increased activity of PFKP (Figures 4D,F). Furthermore, the lactylation modification level of PFKP was linearly negatively correlated with EF (Figure 4G), These findings indicate that the Kla modification of PFKP influences its activity and PFKP-Kla can serve as a potential biomarker for myocardial ischemia.

To identify the lactate modification site of PFKP, we transfected AC16 cells with an adenovirus encoding PFKP (Figure 4H), then enriched PFKP protein via immunoprecipitation followed by mass spectrometry (Figure 4I). Our findings revealed that the sole lactate modification site of PFKP is K688 (Figure 4J). The XIC area in mass spectrometry denotes the peak area corresponding to a specific peak in the Extracted Ion Chromatogram (XIC), a crucial parameter for both qualitative and quantitative analysis. The magnitude of the XIC area directly correlates with the concentration of the target compounds. Analysis of the XIC area indicated an increase in the Kla of PFKP-K688 site during hypoxia and Nala addition, with a subsequent decrease following DCA stimulation (Figure 4K). These results suggest that K688 is a pivotal modification site in PFKP, potentially influencing its functional role under hypoxic conditions.

### 3.5 Lactylation modification of PFKP enhances glycolysis and reduces aerobic respiration

**FIGURE 5:**
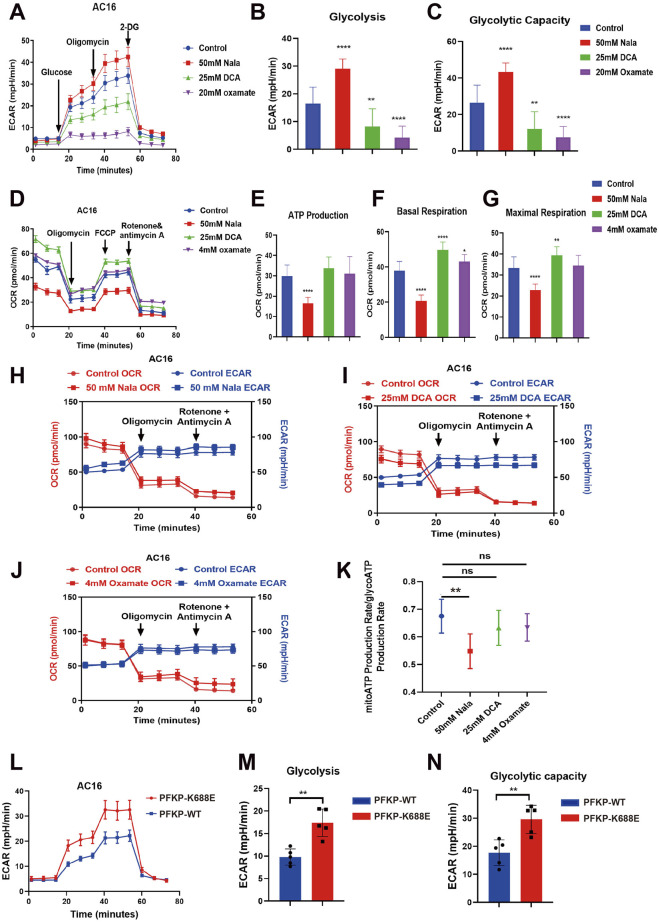
Detection of cell metabolism mode. **(A–J)** Detection of glycolytic ECAR and mitochondrial oxidative phosphorylation OCR (n = 6). **(K)** Mitochondrial/glycolytic ATP production rate (n = 6). **(L–N)** ECAR detection of PFKP-WT and K688E mutations (n = 5). Statistical significance was calculated using one-way ANOVA. Data are shown as mean ± SD. ****P < 0.0001; **P < 0.01; *P < 0.05, ns means no significance.

We investigated the function of PFKP-Kla by analyzing changes in ECAR and OCR following the addition of Nala, DCA, and oxamate to manipulate cellular lactate levels, thereby influencing lactylation of PFKP (Figures 5A–J). Our experimental findings demonstrated that enhancing Kla modification of PFKP in AC16 cells by adding Nala increased ECAR and significantly increased glycolysis compared with the Control group. Conversely, DCA and oxamate (LDH inhibitors) suppressed lactate production, resulting in a significant decrease in glycolytic capacity (Figures 5A–C). Furthermore, the OCR assay revealed that Nala hindered aerobic respiration, ATP production, basal respiratory rate, and maximal respiratory capacity, while the opposite effect was observed following DCA and oxamate treatment (Figures 5D–G). The ratios of mitochondrial aerobic respiration and ATP production derived from glycolysis were reduced by Nala treatment but increased after DCA and oxamate administration (Figure 5K). In conclusion, the Kla modification of PFKP influences cellular energy metabolism, with high lactate modification of PFKP boosting glycolysis. To investigate the role of PFKP-K688la, we mutated the K688 site of PFKP to E to simulate hyper-lactylation. We observed that the K688E mutation amplified ECAR levels and enhanced glycolysis compared to the wild type (Figures 5L–N). Taken together, PFKP-K688la contributes to ameliorating hypoxic cardiomyocyte injury and is a key regulatory target.

### 3.6 Increasing PFKP-Kla levels improves hypoxic injury in cardiomyocytes

**FIGURE 6:**
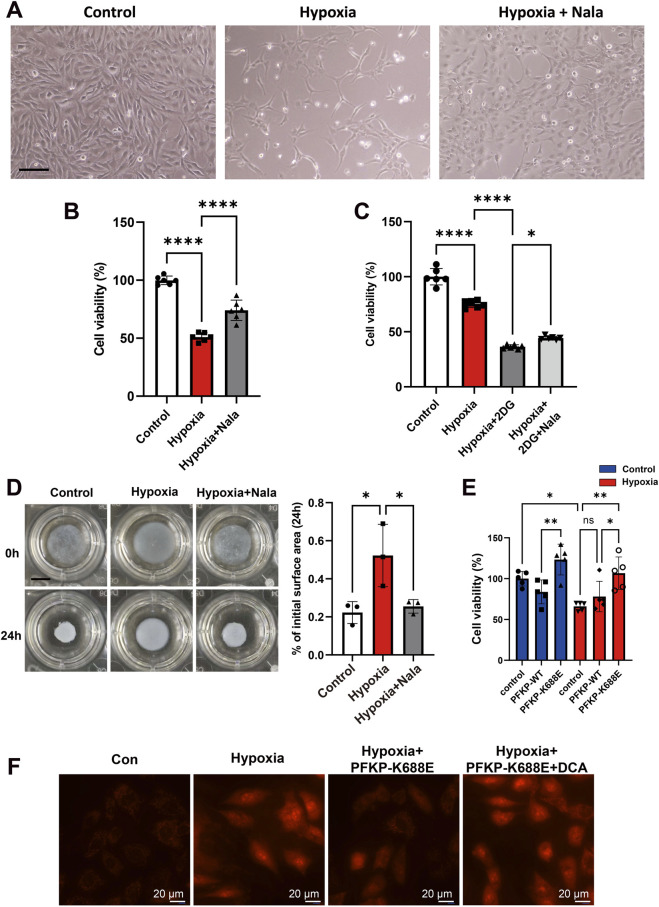
Nala protects the myocardium against hypoxia injury. **(A)** Morphological examination of cardiomyocytes stimulated by Nala (n = 3). Scale bar, 200 μm. **(B,C)** Cell survival test (n = 6). **(D)** Collagen gel contraction test (n = 3). Scale bar, 0.5 mm. **(E)** Cell survival rate assay (n = 5). **(F)** MitoSox assay for reactive oxygen species in mitochondria (n = 3). Statistical significance was calculated using an unpaired two-tailed Student’s t-test or one-way ANOVA. Data are shown as mean ± SD. ****P < 0.0001; **P < 0.01; *P < 0.05, ns means no significance.

Following hypoxia, AC16 cells exhibited a wrinkled and rounded morphology. The introduction of Nala elongated the morphology of hypoxic AC16 cells, restoring it to its normal state (Figure 6A). Nala supplementation enhanced the survival of AC16 cells under hypoxic conditions (Figure 6B). Conversely, the addition of 2-deoxyglucose (2-DG) reduced cell viability by impeding glycolysis; however, Nala partially counteracted the impact of 2DG (Figure 6C). Moreover, Nala reinstated cell contractility during hypoxia (Figure 6D). The experiment shows that overexpression of PFKP can improve the survival rate of hypoxic cardiac cells, and PFKP-K688E can further enhance the survival rate of hypoxic cardiac cells (Figure 6E). Meanwhile, PFKP-K688E can reduce the ROS in hypoxic myocardium. In contrast, DCA can partially counteract the protective effect of K688E (Figure 6F). These results suggest that enhancing PFKP-Kla level can ameliorate hypoxic injury of cardiomyocytes.

## 4 Discussion

**FIGURE 7:**
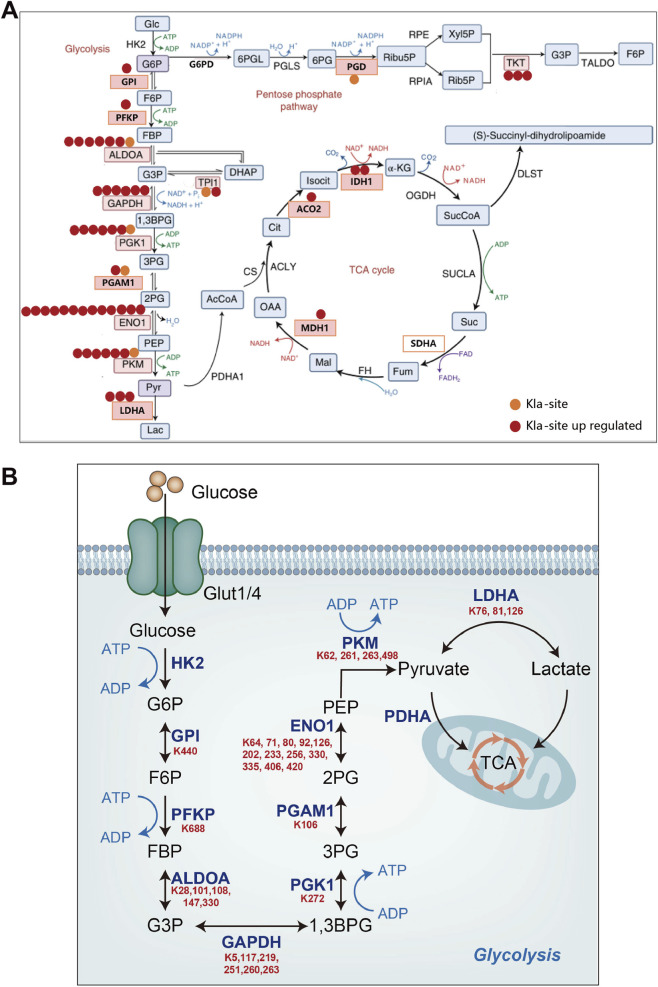
Schematic diagram of the Kla modified protein. **(A)** Changes in the Kla sites of proteins related to glycolytic pathways. **(B)** The specific Kla modification sites in proteins involved in glycolytic pathways.

This study uncovers a novel mechanism in which PFKP serves as a pivotal molecular switch, dynamically regulating cellular energy metabolism reprogramming and influencing cell survival during myocardial ischemia/hypoxia. Initially, myocardial ischemia/hypoxia triggers aerobic respiratory blockade, prompting a compensatory increase in glycolysis and the accumulation of lactic acid, well-known pathophysiological responses. However, our investigation elucidates that the accumulated lactic acid not only serves as a metabolic byproduct or energy source but also impacts cardiomyocyte metabolism and function through the induction of extensive protein lactylation modifications (1053 Kla sites on 521 proteins identified by proteomics). Specifically, these lactylated proteins are significantly enriched in metabolic pathways, especially metabolic enzymes in the glycolytic pathway (Figure 7A). Among the key glycolytic enzymes, both PFKP and PKM undergo lysine lactylation, and notably, only one lactylation site is identified on PFKP (Figure 7B). Given this unique modification profile, we focused our investigation on the K688 lactylation site of PFKP. Notably, we observed that while the expression of PFKP protein remained unaltered under hypoxic conditions, its K688 lactylation modification level significantly rose under hypoxia or stimulation with an exogenous lactate donor (Nala), leading to heightened enzyme activity. Conversely, reducing lactate levels (via DCA or oxamate) inhibited PFKP-K688la and PFKP activity. Functionally, the augmentation of PFKP-K688la directly enhances glycolytic flux while suppressing mitochondrial aerobic respiration, establishing an adaptive feedback loop: hypoxia triggers lactic acid production, which, in turn, amplifies glycolysis by modifying PFKP to expedite energy supply.

Lactate modification drives the progression of metabolic diseases by modulating glycolytic metabolic enzymes. As a signaling molecule, lactic acid regulates glucose metabolism in various cell types by histone lactylation (Jiang et al., 2021; Pan et al., 2022). For instance, the modification of histone H4K12la increases the expression of glycolysis- and inflammation-related genes, such as HIF-1α, in Alzheimer’s disease microglia, thereby enhancing glycolysis (Pan et al., 2022). Conversely, in non-small cell lung cancer, histone lactylation suppresses hexokinase expression while increasing tricarboxylic acid cycle enzyme expression, leading to inhibition of glycolysis (Jiang et al., 2021). Furthermore, the accumulation of lactic acid can directly inhibit the activity of glycolysis upstream enzymes through lactate modification, causing feedback inhibition (Wan et al., 2022).

Key rate-limiting enzymes in glycolysis comprise hexokinase (HK), phosphofructokinase 1 (PFK-1), and pyruvate kinase (PK) (Ritov and Kelley, 2001; Xu et al., 2021). PFK-1 is present in three isoforms: PFKP (platelet), PFKM (muscle), and PFKL (liver) (Li et al., 2018). Studies have demonstrated that PFKP can be phosphorylated and modified by lactate. For instance, in normoxic and hypoxic conditions, numerous non-histone lactate modifications have been observed in oral squamous cell carcinoma (OSCC). Specifically, the K688 site of PFKP has been identified as undergoing lactate modification, suggesting a potential positive feedback loop of hypoxia-glycolysis-Kla modification in OSCC (Song et al., 2024). However, the precise function of this modification remains unclear. Intriguingly, proteomic analysis revealed lactated PFKP in SW480 colon cancer cells (Cheng et al., 2023), with the level of modification showing a negative correlation with enzyme activity, contrary to findings in cardiomyocytes. This discrepancy could stem from variations in microenvironmental pH between tumor cells and cardiomyocytes, or differences in metabolite and cofactor concentrations, necessitating validation through further experiments.

Conventional therapies for coronary heart disease focus on enhancing myocardial oxygen supply or decreasing oxygen demand. Novel therapeutic approaches target enhancing myocardial oxygen utilization efficiency, specifically, by inhibiting fatty acid oxidation and promoting glucose oxidation, myocardial energy efficiency can be significantly enhanced (Lopaschuk, 2003). The PFKP-K688la modification inhibits mitochondrial respiration, potentially reducing energy generation from fatty acid oxidation while augmenting glycolysis, thereby improving cardiac efficiency and optimizing energy substrate selection. This mechanism plays a crucial role in coordinating cardiac energy utilization in hypoxia-ischemia conditions.

The PFKP-K688la-mediated metabolic reprogramming confers a notable protective effect on cardiomyocytes. Augmenting PFKP-Kla levels significantly enhanced the viability of hypoxic cardiomyocytes, reinstated their typical morphology and contractile function, and counteracted the deleterious impacts of the glycolytic inhibitor 2-DG. A K688E point mutation emulates hyper-lactylation and further boosts glycolytic capacity. However, validation of its protective efficacy in physiological settings necessitates assessment in an *in vivo* model. Additional validation of lactylation modification precision can be achieved through the development of antibodies targeting the PFKP-K688 site. The functionality of PFKP-Kla modification can be confirmed by introducing the delactylation-modified K688R mutation to mimic the effects of delactylation modification.

Targeting PFKP-K688la under hypoxic conditions represents a potential therapeutic approach for managing myocardial ischemia. Strategies include: (1) elevating lactate levels through exogenous lactate donors (e.g., Nala) or inhibiting lactate efflux transporters; (2) enhancing the activity or expression of lactate-modifying enzymes (e.g., p300); (3) developing small molecule agonists to activate PFKP-K688la; and (4) selectively upregulating PFKP-K688E expression in the myocardium via gene editing or viral vector delivery systems. Nonetheless, these approaches encounter challenges. The reversible and environment-dependent nature of lactate modification necessitates precise regulation to prevent acidosis or metabolic imbalances from excessive augmentation. Moreover, the scarcity of tools for targeting Kla underscores the critical need for the development of particular small-molecule agonists.

This investigation establishes a connection between lactic acid buildup, a metabolic indicator of ischemia and hypoxia, lactic acid modification, and the regulation of glycolytic enzyme PFKP activity. It elucidates that the lactic acid-PFKP-Kla axis serves as a crucial molecular nexus for myocardial cells to manage energy deprivation, offering a fresh perspective on comprehending the metabolic adaptation mechanisms in ischemic heart disease.

## 5 Conclusion

This study unveils the novel finding that the PFKP-K688la serves as a crucial adaptive mechanism in cardiomyocytes during hypoxic stress. This post-translational modification significantly contributes to the preservation of cellular energy balance and attenuation of hypoxic injury through the augmentation of PFKP activity and glycolytic flux. These insights offer a fresh outlook on the metabolic regulatory pathways implicated in ischemic heart disease and propose lactate-modified PFKP as a promising therapeutic target.

## Data Availability

The mass spectrometry proteomics data have been deposited to the ProteomeXchange Consortium (https://proteomecentral.proteomexchange.org) via the iProX partner repository with the dataset identifier PXD075014. Additional Supplementary Material are available via the article link.
