# Supplementary material for: Lactylation of PFKP-K688 enhances glycolytic flux and confers cardioprotection in myocardial ischemia
Source: Front Pharmacol. 2026 Mar 16;17:1717779. doi: 10.3389/fphar.2026.1717779 (PMC13033730; doi:10.3389/fphar.2026.1717779)

Full unedited gel for Figure 1C

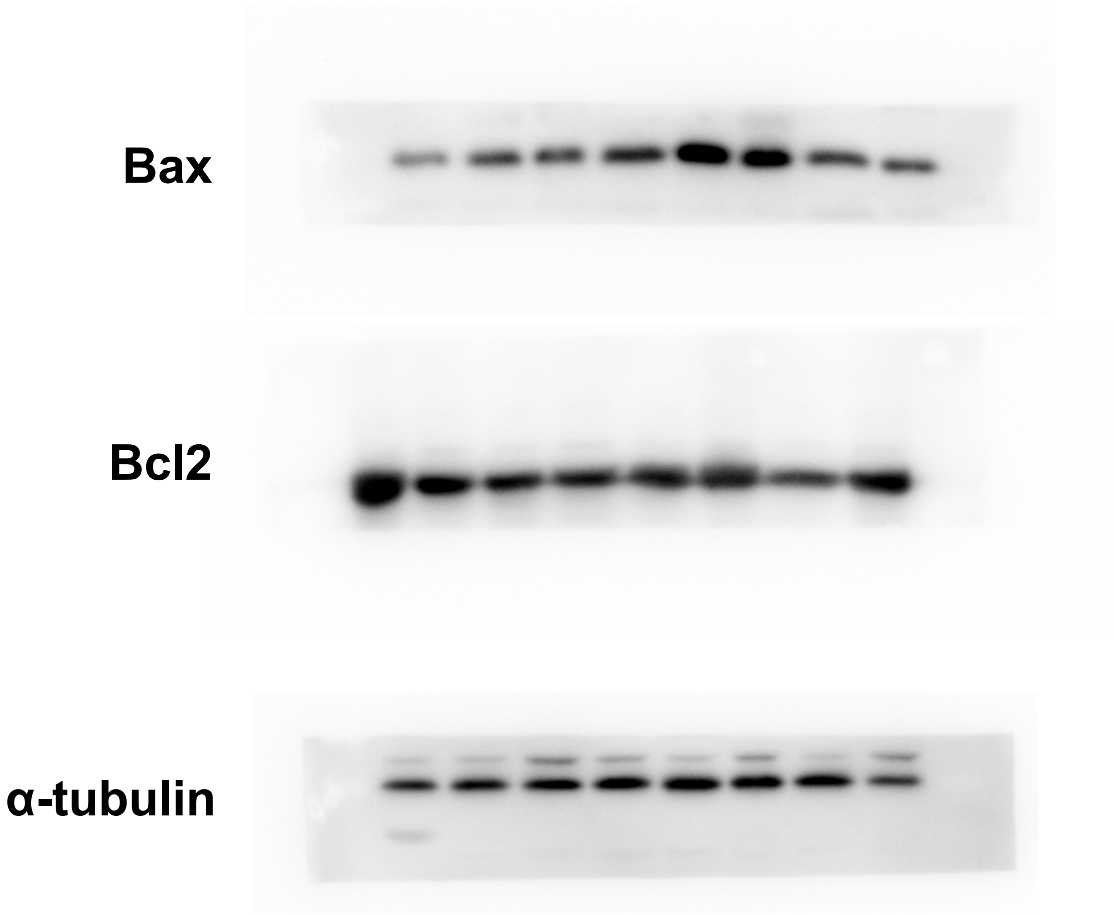

Full unedited gel for Figure 2A

Anti-Kla

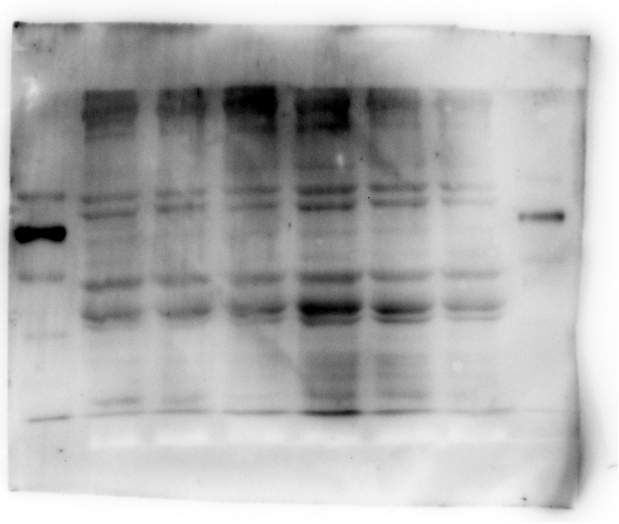

Coomassie Brilliant Blue

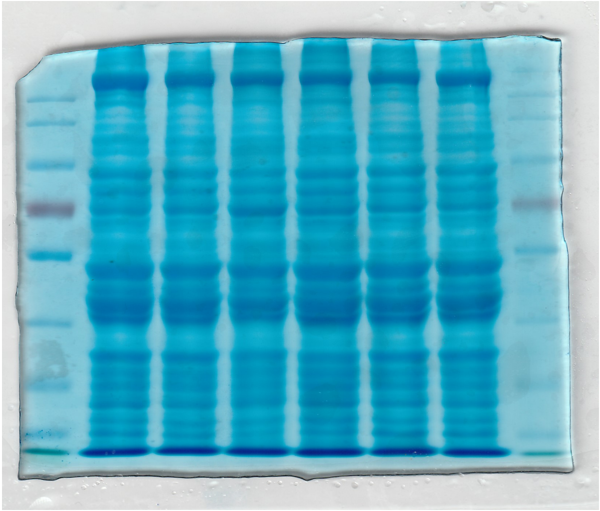

Full unedited gel for Figure 2B

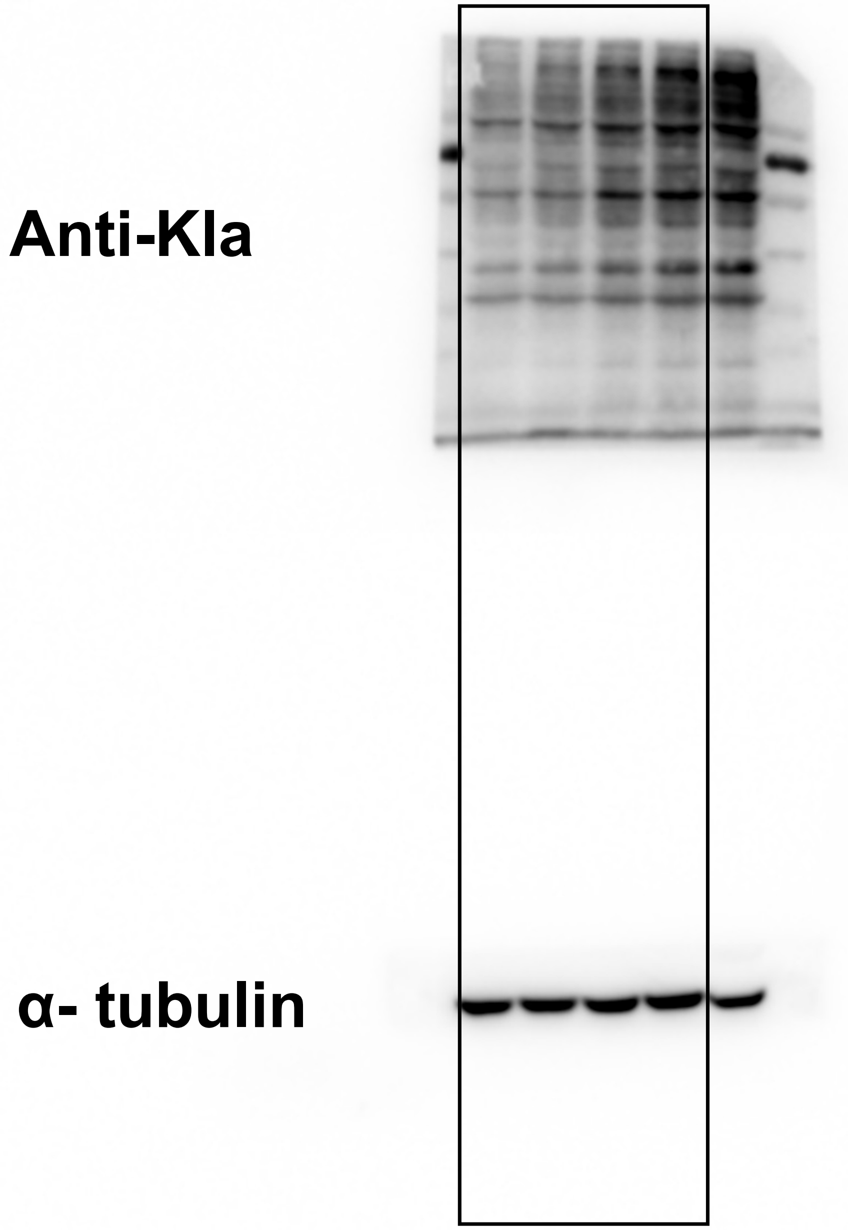

Full unedited gel for Figure 4A

PFKP

α- tubulin

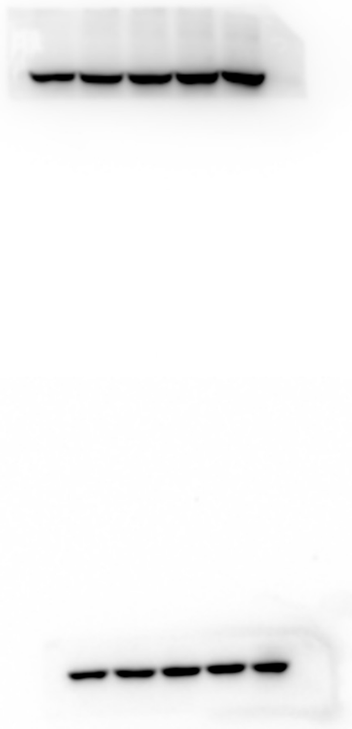

Full unedited gel for Figure 4B

IP

Anti-Kla

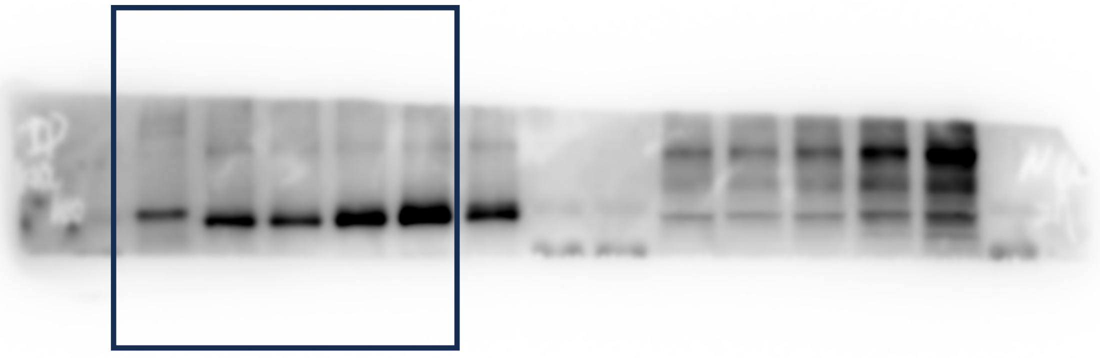

PFKP

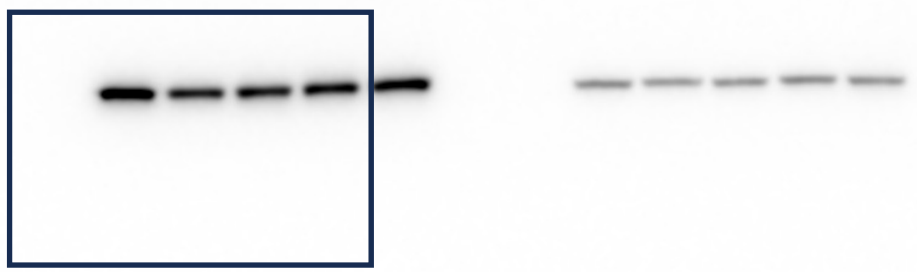

Full unedited gel for Figure 4B

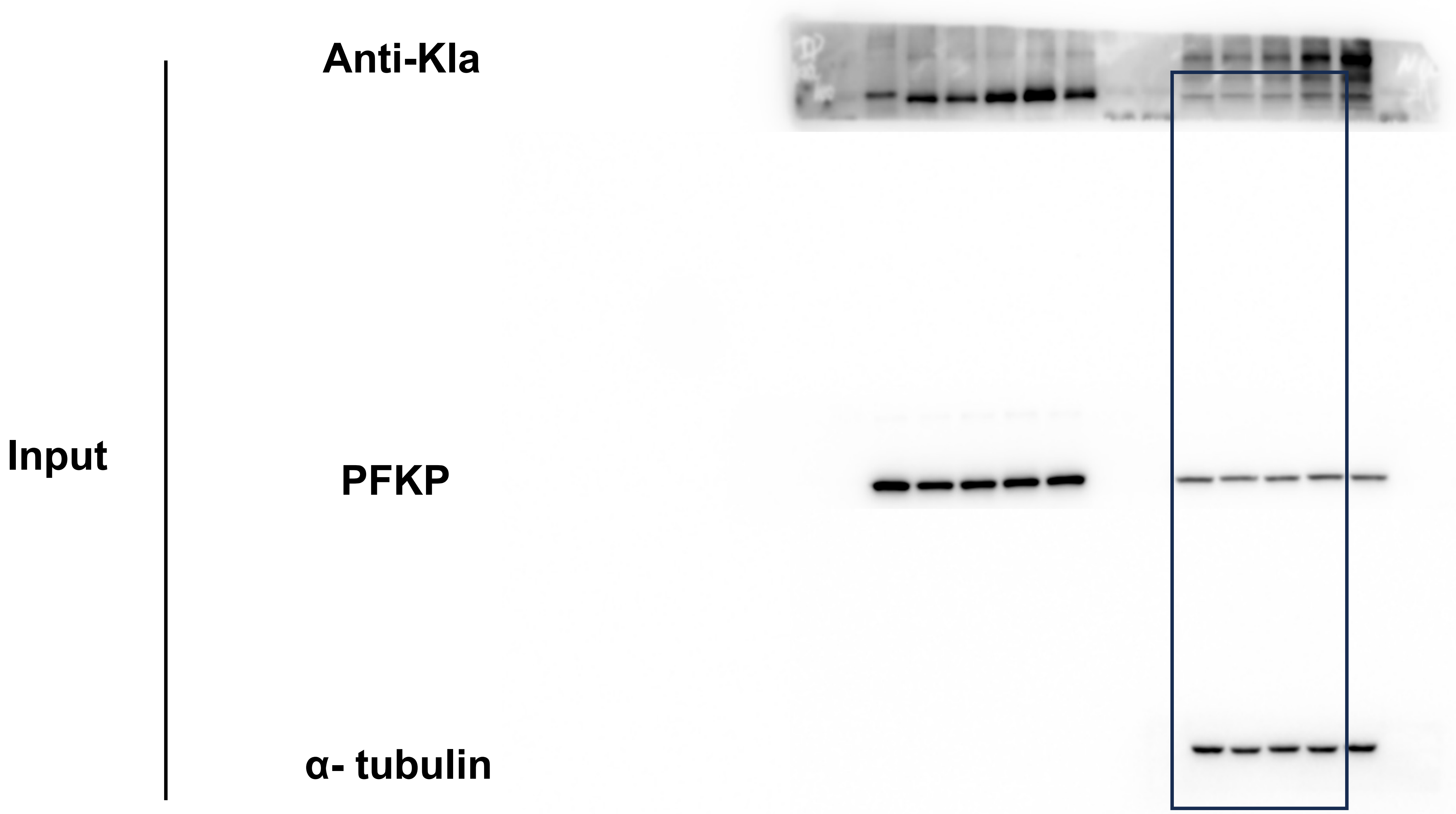

Full unedited gel for Figure 4C （DCA IP）

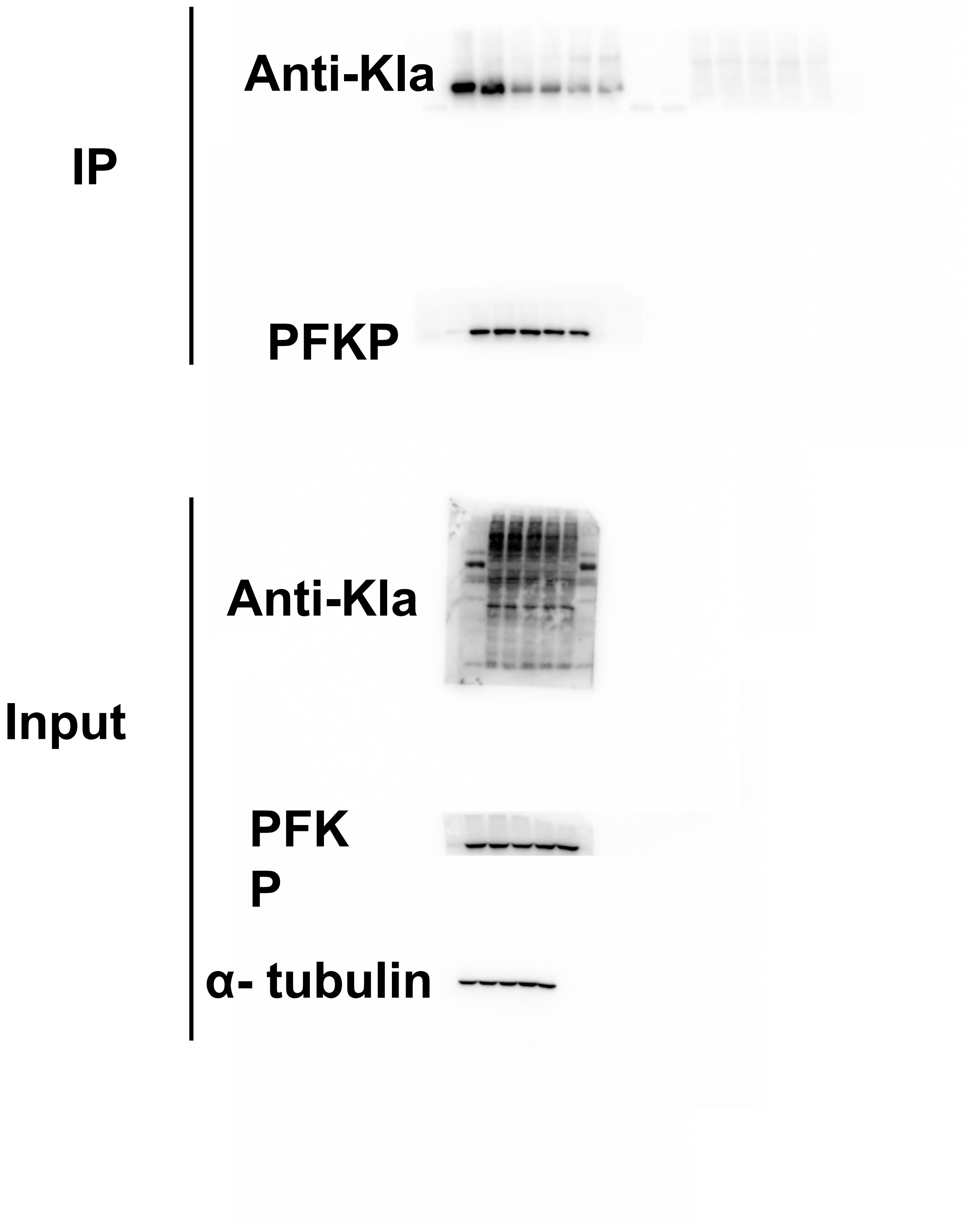

Full unedited gel for Figure 4D

IP

Anti-Kla

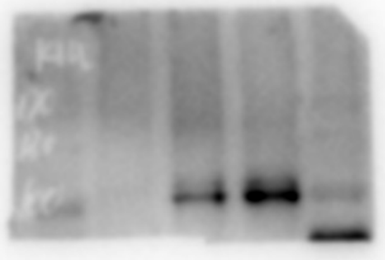

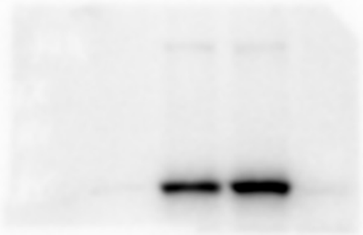

PFKP

Full unedited gel for Figure 4D

Input

Anti-Kla

PFKP

α- tubulin

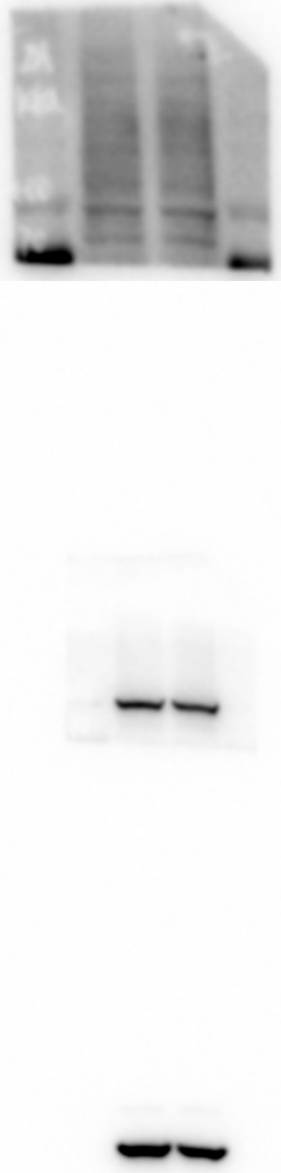

Full unedited gel for Figure 4I

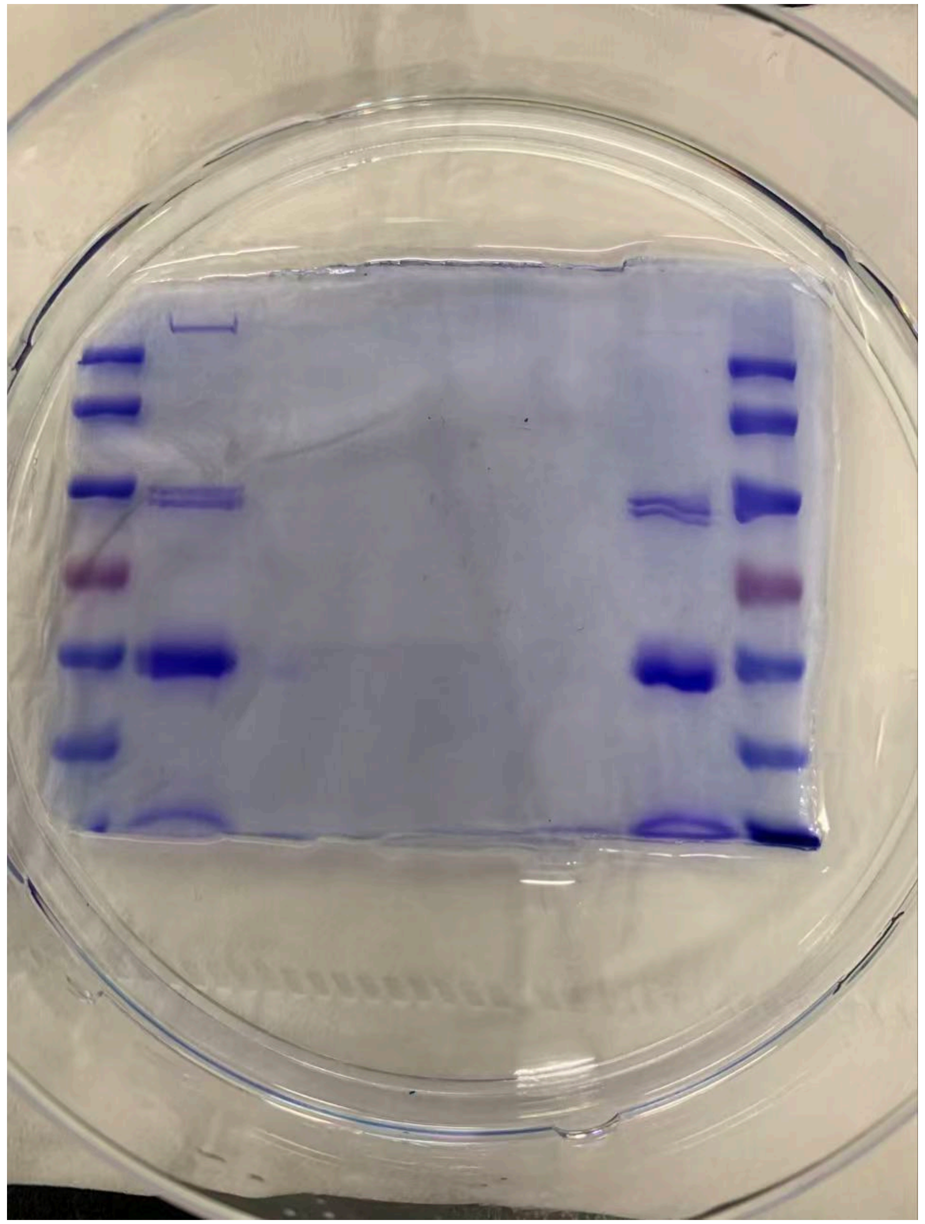

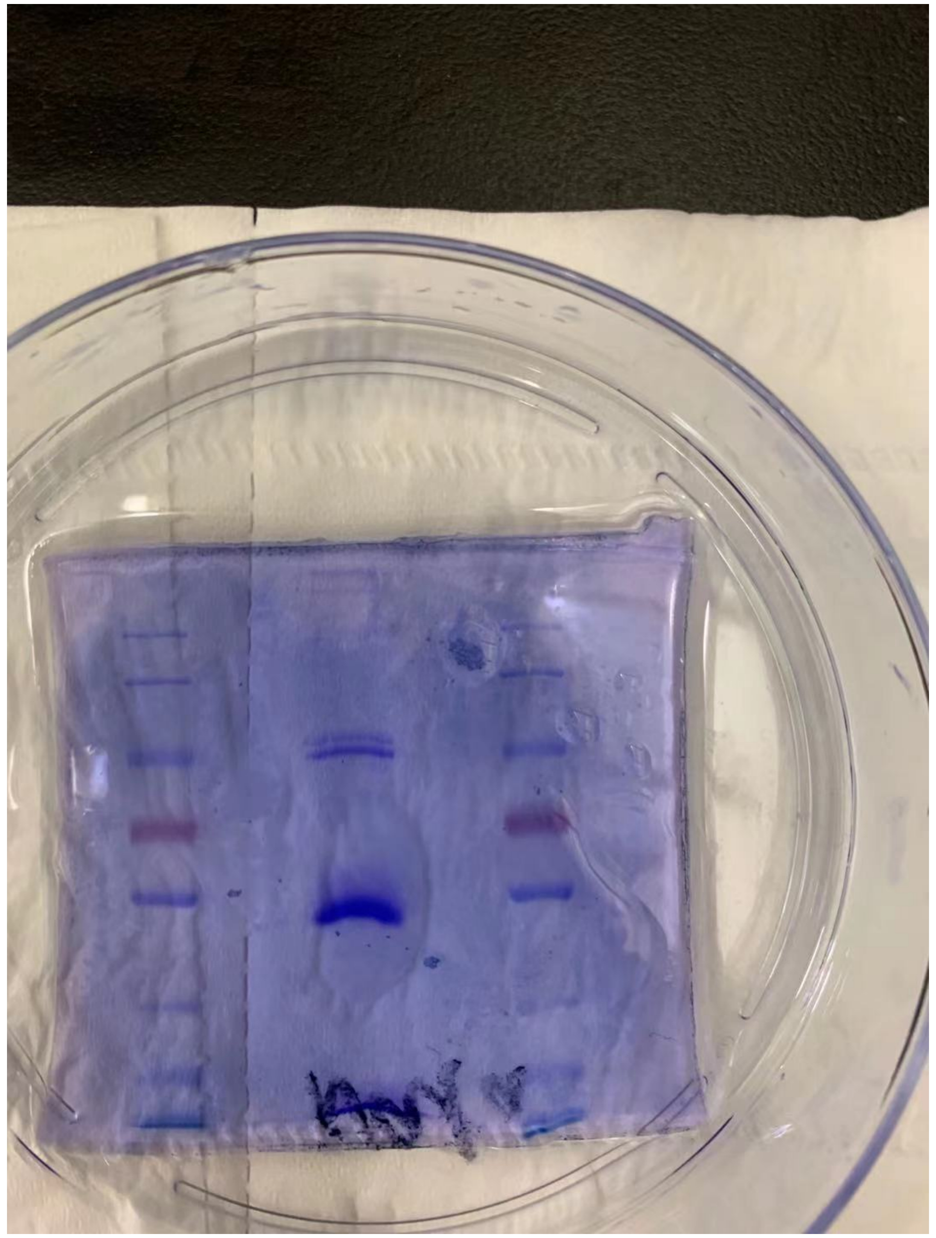

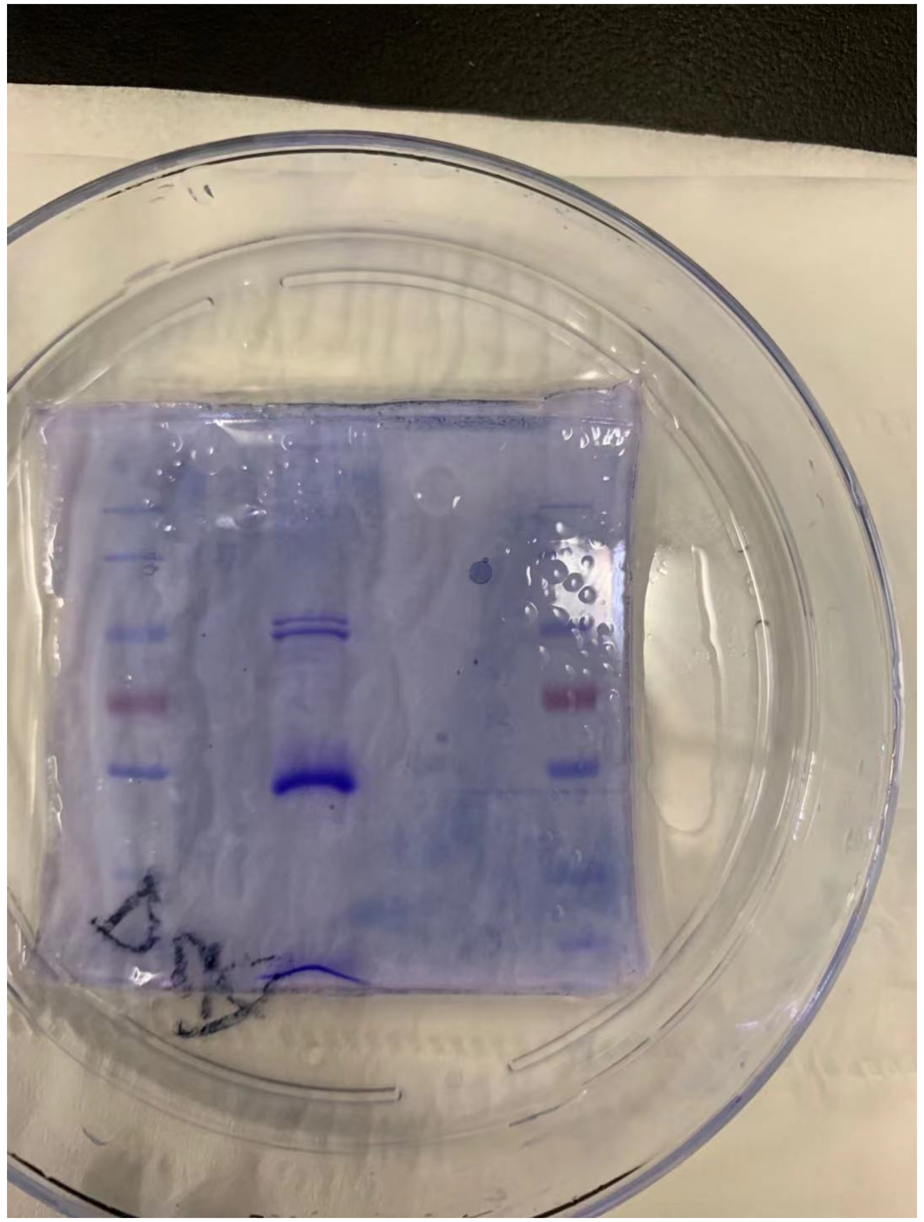

Supplement: Supplementary file 2 [file Supplementaryfile1.pdf]
