# Supplementary material for: Lactylation of PFKP-K688 enhances glycolytic flux and confers cardioprotection in myocardial ischemia
Source: Front Pharmacol. 2026 Mar 16;17:1717779. doi: 10.3389/fphar.2026.1717779 (PMC13033730; doi:10.3389/fphar.2026.1717779)
Supplement: Supplementary file 4 [file Image1.pdf]

Supplementary Material

1 Supplementary figures

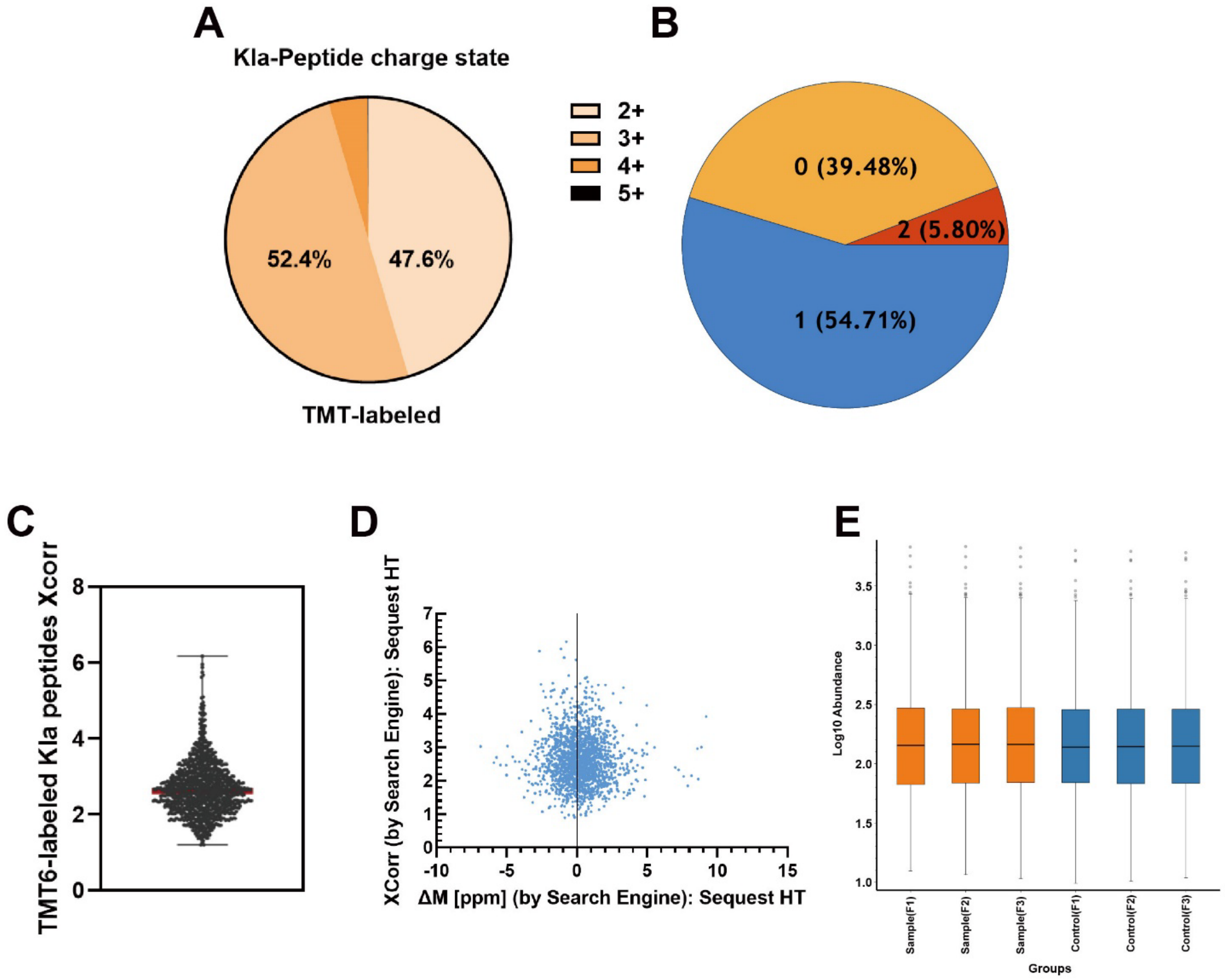

Figure S1. Quantitative proteomics quality control of lactylation-modified TMT-6 in AC16 cells with hypoxia.(A) The charge states of Kla-peptides (B) The missed-cleavage sites of peptides. (C) The Xcorr value of Kla-peptides. (D) The Δ M(ppm) distribution of all fragments. (E) The peptides abundance among different sample.
